# Malignant peripheral nerve sheath tumor as a cause of chronic cardiac insufficiency in cattle

**DOI:** 10.1186/1751-0147-55-7

**Published:** 2013-01-31

**Authors:** Saulo Petinatti Pavarini, Danilo Carloto Gomes, Marcele Bettim Bandinelli, Flademir Wouters, Luciana Sonne, David Driemeier, Cláudio Estêvão Farias da Cruz

**Affiliations:** 1 Departamento de Patologia Clínica, Faculdade de Veterinária, Universidade Federal do Rio Grande do Sul (UFRGS), Porto Alegre, Rio Grande do Sul, Brazil

**Keywords:** Cattle, Immunohistochemical procedures, Neoplasm, Malignant schwannoma

## Abstract

Chronic cardiac insufficiency was associated with a malignant peripheral nerve sheath tumor in a cow. An eight-year-old cow developed a progressive condition (over a period of three months) characterized by an enhanced abdominal volume, reluctance to move, a positive jugular pulse, watery diarrhea and death. At necropsy, moderate subcutaneous edema and an enhanced hepatic lobular pattern were observed. A 23x20x11 cm firm, grayish-white mass adhered to and infiltrated the right atrium. Multiple firm, yellowish-white nodules of 0.5 to 12 cm in diameter were diffusely scattered in the epicardium and parietal pericardium. Histologically, the tumor was poorly circumscribed with foci of infiltration of the myocardium. The neoplastic cells had two major histologic patterns, Antoni types A and B. Within occasional foci, pleomorphic cells with an epithelioid appearance were present in addition to multinucleated cells with periodic acid Schiff (PAS)-positive cytoplasmic globules. Foci of cartilaginous and granular differentiations were interspersed among the neoplastic cells. Multiple vessels presented wall hyalinization and tumoral embolus. Large necrotic foci with mineralization and cholesterol clefts were also observed. Immunohistochemically, the tumor was positive for S100 protein, vimentin and neuron-specific enolase labeling.

## Background

Tumors of the peripheral nervous system are common in humans but comparatively rare in domestic animals, having been mostly reported in cattle, dogs, cats and horses [1-4]. Peripheral nerve sheath tumors (PNST) compose a heterogeneous group of neoplasms that includes schwannomas (neurilemomas), neurofibromas and perineuromas. These neoplasms may originate from Schwann cells, fibroblasts, perineural cells, or combinations thereof. In domestic animals, the distinction between schwannomas and neurofibromas is not clearly defined; therefore, both of these are classified as PNST according to the World Health Organization. Based on the morphology and biological behavior, PNST’s may be classified as benign or malignant [5,6]. This type of neoplasm may occur in any location in the peripheral nervous system. In cattle, PNST are often found in autonomic nerves such as those from the epicardial and mediastinal plexus and from the thoracic and cervical sympathetic ganglia [1]. In cattle, PNST are generally asymptomatic and considered to be incidental findings, mainly at the slaughter lines [7-9]. Only a few bovine PNST’s have been associated with clinical disease, and these have usually been linked to compression secondary to the adjacent tumor [10-13]. This paper describes the clinical, pathological and immunohistochemical findings recorded in a case of chronic cardiac insufficiency due to a peripheral nerve sheath malignant tumor infiltrating the heart of a cow.

## Case presentation

**Figure 1:**
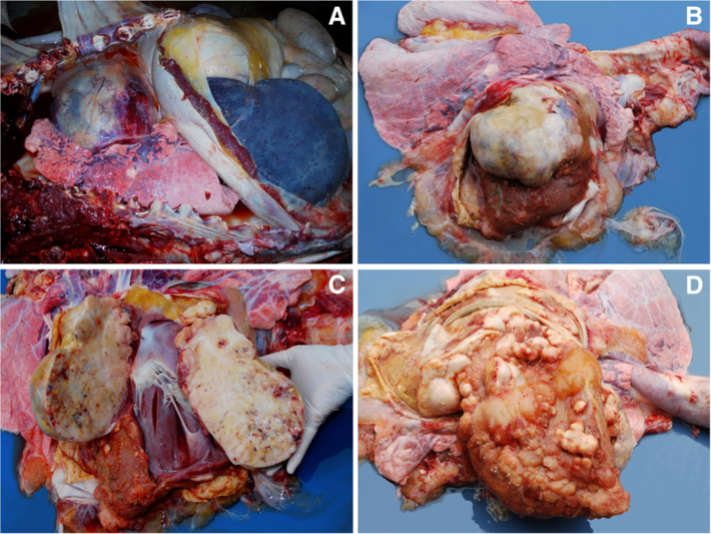
**Bovine cardiac peripheral nerve sheath malignant tumor. **Gross changes. (**A**) Cardiomegaly, with dorsal pulmonary compression. Enhanced liver with rounded edges and blue surface. (**B**) Large grayish-white neoplastic mass adhered to and infiltrating the right atrium. (**C**) Cut surface of the neoplastic mass bearing yellowish areas and hemorrhagic foci. (**D**) Multiple yellowish-white nodules adhered to the visceral pericardium from the left chambers and to the parietal pericardium.

An eight-year-old Holstein cow from a farm located in southern Brazil developed progressive (over the course of three months) abdominal distension. The clinical signs included engorged jugular veins with a positive venous pulse, stiff gait and reluctance to move. Subsequently, the cow developed watery diarrhea and after 13 more days, developed stenosis of the rectum, hypothermia (36°C), prolonged sternal recumbency and then died. At necropsy, there was moderate subcutaneous edema in the ventral neck and sternum. There were 35 and 10 liters of translucent, yellowish liquid (transudate) in the abdominal and thoracic cavities, respectively. The markedly enlarged heart compressed and displaced the pulmonary parenchyma to the dorsum of the thorax (Figure 1A). Four liters of a low-viscosity, reddish fluid was found inside the pericardial sac. A 23x20x11 cm firm, grayish-white mass adhered to and infiltrated the right atrium (Figure 1B). Hemorrhagic foci and yellowish areas with a calcareous consistency (Figure 1C) were observed after cutting the mass. Multiple firm, yellowish-white nodules of 0.5 to 12 cm in diameter were diffusely adhered to the epicardium and parietal pericardium (Figure 1D). The liver had a markedly enhanced volume, rounded edges, and a bluish capsular surface (Figure 1A). Upon cutting, the liver was mildly firm and showed an enhanced lobular pattern. Intense mesocolic and abomasal edema was also present.

### Histopathology

Tissue fragments were sampled, fixed in 10% buffered formalin and routinely processed for histopathology. The tissue sections were stained by the hematoxylin and eosin (H&E) and periodic acid Schiff (PAS) methods. The tumor samples were subjected to streptavidin-biotin-peroxidase immunohistochemistry. Table 1 shows the primary antibodies used and the protocols applied. The secondary rabbit antibody was biotinylated (Dako^®^ Denmark A/S, Glostrup, Denmark) and followed by streptavidin peroxidase (Dako^®^). The 3´3-diaminobenzene (Dako^®^) was used as the chromogen and the samples were counterstained with Harris hematoxylin. The positive controls consisted of samples taken from the central nervous system (GFAP, NSE and neurofilament), peripheral nervous system (S100), skin (cytokeratin) and skeletal muscle (vimentin and desmin) of normal cattle. The negative controls consisted of tumor sections and tissue fragments incubated in phosphate buffered saline (PBS) instead of the primary antibody.

**Table 1:** Primary antibodies and immunohistochemical protocols applied in the study

***Antibody***	***Code no.***	***Clone***	***Dilution***	***Antigen retrieval***
Monoclonal				
Mouse anti-vimentin^b^	180052	V9	1:200	3 min/125°C, 0,01M citrate buffer pH 6.0
Mouse anti-human neuron-specific enolase (NSE) ^a^	M 0873	BBS/NC/VI-H14	1:100	3 min/125°C, 0,01M citrate buffer pH 6.0
Mouse anti-human cytokeratin^a^	M3515	AE1/AE3	1:80	3 min/125°C, 0,01M citrate buffer pH 6.0
Mouse anti-human desmin^a^	M 0760	D33	1:300	Microwave (700w), 3x 5min,0,01M citrate buffer pH 6.0
Polyclonal				
Rabbit anti-S100^a^	Z 0311		1:200	20 min/100°C, 0,01M citrate buffer pH 6.0
Rabbit anti-glial fibrillary acidic protein (GFAP) ^a^	Z 0334		1:500	10 min/100°C, Tris-EDTA buffer Ph 9,0
Rabbit anti-bovine neurofilament^c^	AHP245		1:500	10 min/37°C Trypsin 0,1% and Microwave (700w), 2 min, 0,01M citrate buffer Ph 6.0
Rabbit anti-human Von Willebrand Factor^a^	A0082		1:800	3 min/125°C, 0,01M citrate buffer Ph 6.0

^a ^Dako Denmark A/S, Glostrup, Denmark.

^b^Zymed Laboratories Inc., San Francisco, USA.

^c^AbDSerotec, Oxford, UK.

**Figure 2:**
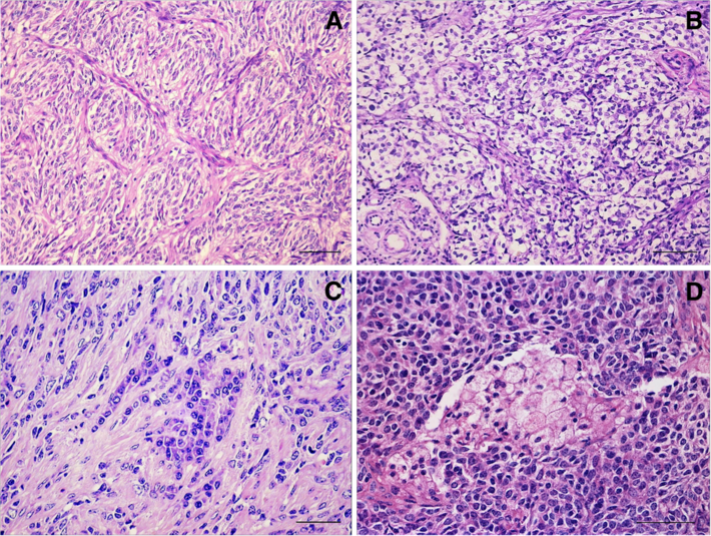
**Histopathological findings from the peripheral nerve sheath malignant tumor in the heart of a cow. **(**A**) Dense fusiform cell proliferation arranged in short bundles in several directions and forming irregular nests bordered by fibrovascular stroma (type A Antoni pattern). Bar = 90 μm, H&E. (**B**) Proliferation of loose, round-to-oval-shaped neoplastic cells with indistinct edges and surrounded by a mildly basophilic myxoid matrix (type B Antoni pattern). Bar = 100 μm, H&E. (**C**) Neoplastic cells with an epithelioid appearance. Bar = 70 μm, H&E. (**D**) Proliferation focus of granular cells. Bar = 50 μm, H&E.

Histologically, the tumor was poorly circumscribed with foci infiltrating the myocardium. Most of the neoplastic cells had two histologic patterns. High-density fusiform or slightly oval-shaped cells were oriented in short bundles at various locations and occasionally intertwined in spiral or palisade patterns, forming irregular nests delimitated by the fibrovascular stroma (type A Antoni) (Figure 2A). These cells had indistinct, mildly eosinophilic cytoplasms. The nuclei were slightly fusiform with loose chromatin, and most had a single evident nucleolus. The second histologic pattern observed in another distinct area was characterized by the proliferation of loose, round- to-oval-shaped cells with indistinct cellular edges and wrapped by varying amounts of a myxoid matrix (type B Antoni) (Figure 2B). The nuclei were rounded with condensed chromatin and indiscernible nucleoli. Occasional foci contained polygonal cells arranged in irregular cords, with well-delimitated eosinophilic and abundant cytoplasms, giving the cells an epithelioid appearance (Figure 2C). These cells were pleomorphic and contained large nuclei with varied forms, coarse chromatin, and evident, sometimes multiple, nucleoli. Multinucleated cells with intracytoplasmic PAS-positive globules were also observed in addition to areas with granular, large, round- to-polygonal-shaped cells. These cells had well-defined edges, abundant cytoplasms and finely vacuolated, amphophilic (PAS-positive) and distinct nuclei, which were sometimes displaced to the cellular periphery with a prominent and magenta nucleolus (Figure 2D). Among the neoplastic cells, there also were multifocal areas of cartilaginous differentiation. Mitotic figures were also observed sporadically (an average of 2 per field at 400X magnification). Wall hyalinization was evident in multiple small and large vessels apart from intraluminal groups of neoplastic cells. Numerous areas of necrosis with mineralization and cholesterol clefts were associated with extensive hemorrhage surrounded by hemosiderin-laden macrophages. Severe sinusoidal congestion was associated with hepatocyte loss and centrolobular fibroplasia.

**Figure 3:**
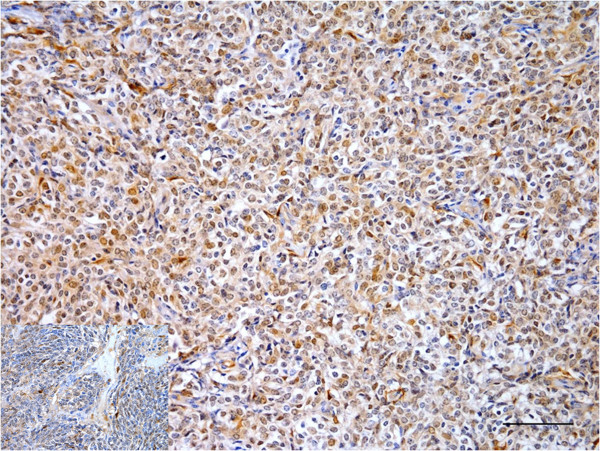
**Marked nuclear and cytoplasmic anti-S100 immunolabeling in the PNST from a cow. **Bar = 100 μm. Inset: immunostained neoplastic cells within a blood vessel.

**Figure 4:**
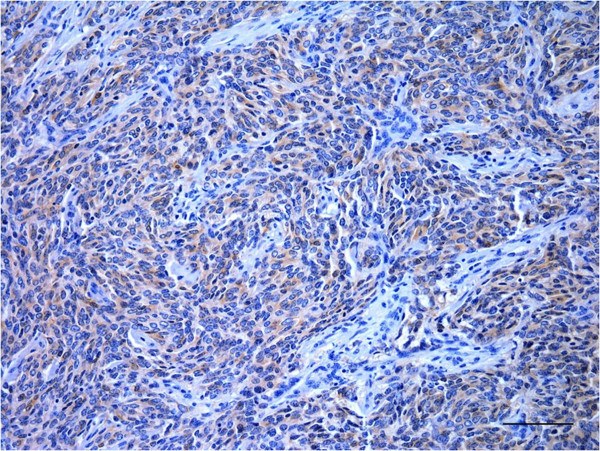
**Moderate diffuse cytoplasmic anti-NSE immunolabeling in the PNST from a cow. **Bar = 100 μm.

**Figure 5:**
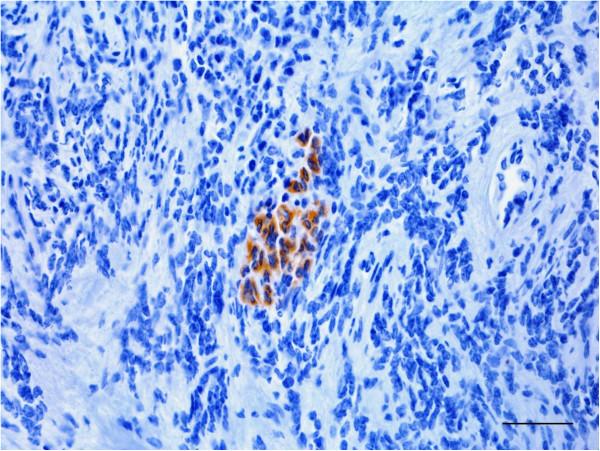
**Focal cytoplasmic anti-cytokeratin immunolabeling in cells with an epithelioid resemblance. **PNST in a cow. Bar = 60 μm.

Multifocal areas of moderate to marked anti-S100 protein immunolabeling (Figure 3) were observed in the cytoplasm and nuclei of the neoplastic cells. Diffuse, moderate and predominantly cytoplasmic anti-vimentin immunolabeling in addition to multifocal, moderate and cytoplasmic anti-NSE immunostaining (Figure 4) and differentiated epithelioid cells positively stained for cytokeratin (Figure 5) were also observed; however, there was no reactivity of the tumoral cells with additional antibodies tested (specific for GFAP, desmin, von Willebrand factor and neurofilament).

## Discussion and conclusions

The diagnosis of chronic cardiac insufficiency caused by a malignant peripheral nerve sheath tumor in the heart of a cow was based on findings such as type A and B Antoni patterns and immunolabeling (anti-vimentin, anti-S100, and anti-NSE), all of which are consistent with a PNST, specifically a schwannoma [12,14-16]. The histological differentiation between malignant and benign PNST can be difficult because both may show undefined edges and some degree of cellular pleomorphism [17]. It has been suggested that malignant PNST in cattle have invasive areas in the adjacent tissue, extensive necrotic areas and cellular pleomorphism [12,18,19]. All of these characteristics were observed in this case; however, the presence of neoplastic cells within the blood vessels was the main finding that determined the classification of malignancy. A high level of mitosis is also indicative of a malignant PNST [12,19], but this finding may be absent [17], as was the case here. Granular and cartilaginous differentiations were observed in the neoplasm. In addition, schwannomas may also present bone, glandular and melanotic differentiations [20-24], because migratory cells from the neural crest can differentiate into melanocytes and Schwann, ganglionic and mesenchymal cells, which contribute to form muscle, bone and cartilage in the head and neck [20]. In dogs and humans, divergent differentiation is usually associated with a poor prognosis [24,25]. The S100 protein is the primary marker in the diagnosis of bovine PNST (schwannoma and neurofibroma) and may be used as a single diagnostic tool [13,26-28] or in combination with other markers such as GFAP and NSE [7,17,19,29,30]. The neoplastic cells in this study showed multifocal positivity for S100 and NSE immunolabeling but were negative for GFAP. Not all neoplastic cells from occasional human malignant schwannomas demonstrate anti-S100 immunolabeling due to the particular differentiation stages of the nervous cells [31]. Anti-GFAP immunolabeling is a characteristic more commonly found in benign than malignant PNST in the dog [20]. Anti-cytokeratin immunolabeling is usually not observed in cases of bovine PNST [15], but it was observed in cells with an epithelioid pattern in the case reported here. Anti-cytokeratin immunolabeling has been associated with occasional malignant schwannomas, especially when the primary antibody is a pancytokeratin marker (AE1/AE3) [16]. Multifocal distributions of malignant PNST in cattle have been described [13,26,27,30]. It is believed that multicenter schwannomas result from a simultaneous neoplastic transformation, rather than a metastatic process derived from a single primary site [15,27]. However, other authors suggested that the neoplastic cells may disseminate from a primary focus to other organs through metastasis [32]. In the present tumor, multiple small nodules were detected in the visceral and parietal pericardium, in addition to a large mass adhering to and infiltrating the right atrium and associated with large numbers of neoplastic cells inside the blood vessels. Such tumoral emboli indicate the possibility that metastasis might have occurred from the tumoral mass.

PNST in cattle are usually incidental findings upon necropsy or slaughter [7-9]. Clinical manifestations in cattle affected by PNST are sporadic and include limited mobility caused by limb paresis or paralysis, cranial nerve- and brainstem-related disorders, vagal indigestion, dyspnea, recurrent ruminal bloat, and progressive wasting [10-13,18,26,33]. The cow in this study showed clinicopathological chronic cardiac insufficiency expressed by a reluctance to move, engorged jugular veins with a positive pulse, cavity edema, and an enhanced hepatic pattern, clinical manifestations caused by the tumor expansion, which compressed the heart and prevented adequate cardiac output. Subsequent events included venous stasis and an increase of the hydrostatic pressure in the blood vessels. It has been suggested that because they are slow-growing neoplasms, PNST are common in old cattle [12]. Information presented here is useful for the differential diagnosis of the bovine chronic cardiac failure.

## Competing interests

The authors declare that they have no competing interest with respect to their authorship or the publication of this article.

## Authors’ contributions

SPP retrieved clinical information and with the help of DCG, MBB and FW carried out necropsy, histopathology and immunohistochemical studies. LS, DD and CEFC prepared the manuscript, including figures and revisions. All authors have read and approved the final version of the manuscript.
